# Mapping Thyroarytenoid and Cricothyroid Activations to Postural and Acoustic Features in a Fiber-Gel Model of the Vocal Folds

**DOI:** 10.3390/app9214671

**Published:** 2019-11-01

**Authors:** Anil Palaparthi, Simeon Smith, Ingo R. Titze

**Affiliations:** 1 National Center for Voice and Speech, The University of Utah, 1901 S Campus Dr, Suite 2120, Salt Lake City, UT 84112, USA; 2 Department of Bioengineering, The University of Utah, Salt Lake City, UT 84112, USA

**Keywords:** intrinsic laryngeal muscle activations, voice acoustics, vocal fold posturing, voice production

## Abstract

Any specific vowel sound that humans produce can be represented in terms of four perceptual features in addition to the vowel category. They are pitch, loudness, brightness, and roughness. Corresponding acoustic features chosen here are fundamental frequency (*f_o_*), sound pressure level (SPL), normalized spectral centroid (NSC), and approximate entropy (ApEn). In this study, thyroarytenoid (TA) and cricothyroid (CT) activations were varied computationally to study their relationship with these four specific acoustic features. Additionally, postural and material property variables such as vocal fold length (L) and fiber stress (*σ*) in the three vocal fold tissue layers were also calculated. A fiber-gel finite element model developed at National Center for Voice and Speech was used for this purpose. Muscle activation plots were generated to obtain the dependency of postural and acoustic features on TA and CT muscle activations. These relationships were compared against data obtained from previous in vivo human larynx studies and from canine laryngeal studies. General trends are that *f_o_* and *SPL* increase with CT activation, while NSC decreases when CT activation is raised above 20%. With TA activation, acoustic features have no uniform trends, except SPL increases uniformly with TA if there is a co-variation with CT activation. Trends for postural variables and material properties are also discussed in terms of activation levels.

## 1. Introduction

This study was motivated by a desire to eventually control a voice simulator with inputs related to perception. Aside from vowel perception, a sound can be represented in terms of pitch, loudness, and timbre [1]. Timbre is the quality of the sound that differentiates one sound from another when pitch and loudness are the same [2]. In a first attempt, it can be divided into two components, brightness and roughness [3]. Acoustically, the four perceptual features can be quantified with fundamental frequency, sound pressure level, spectral content, and aperiodicity. Neglecting additive noise from air turbulence (breathiness or aspiration in a vowel), it is believed that these four perceptual or acoustic features contain many of the characteristics of a given vowel sound [4]. Quantifying fundamental frequency and sound intensity is well described in the literature. The fundamental frequency of a sound can be measured directly from the oral pressure signal using standard algorithms such as autocorrelation, zero-crossing rate or with more advanced algorithms such as the Yin algorithm [5] or SWIPE’ [6]. Sound pressure level, the logarithmic expression of relative sound intensity, can be computed from the radiated pressure signal according to acoustic standards [7]. Brightness is much more abstract and has no agreed-upon standards for calculation on the acoustic signal. Some acoustic features that represent brightness are the total harmonic distortion [8] and the normalized spectral centroid [2,9]. Roughness also has no standard definition in the literature other than it being attributed to the component of the sound that is not regular (periodic) [10,11]. However, even periodic sounds can be perceived as rough if multiple harmonics are contained in a critical band [12]. Measures of aperiodicity roughness are jitter and shimmer [13], while a measure of lack of roughness is the cepstral peak prominence (CPP) [14–16]. Non-linear dynamic metrics such as correlation dimension [17,18] and various entropy measures [18,19] are also being studied to accurately quantify aperiodicity. In this study, roughness is considered to be related to the power in the sound that is not part of the fundamental and its harmonic series. Subharmonics and sideband frequencies are included in the quantification of roughness.

If the vocal tract configuration (vowel) is kept constant during phonation, all variations in the four perceptual features depend on changes in the vocal source, but the interaction with the vocal tract may vary with fundamental frequency [20]. The laryngeal configuration, and specifically the vocal fold posture, depend on the laryngeal muscle activations [21]. Vocal fold oscillation then depends on the amount of lung pressure available to set the vocal folds into motion. In this study, we focused on the interrelationship between the thyroarytenoid (TA) and cricothyroid (CT) muscle activations to achieve these four specific physical attributes of a sound as well as vocal fold posturing (setting) features. While our computational model includes postural mechanics derived from five intrinsic muscles (lateral cricoarytenoid (LCA), interarytenoid (IA), and posterior cricoarytenoid (PCA) in addition to CT and TA), preliminary results showed that random combinations of five muscle activations and lung pressure produced too complex relationships to comprehend easily. Hence, a heuristic approach was taken to constrain the LCA, IA, PCA, and lung pressure (P_L_) for the purpose of targeting self-sustained oscillation. Future studies with computational learning strategies will address the more intricate relations with all the muscle activations.

The vocal tract configuration remained constant throughout the phonations (an/ɑ/vowel). The posturing variables included are vocal fold length (L) and tissue fiber stress in the superficial layer of the lamina propria (*σ_SLLP_*), ligament (*σ_lig_*), and TA muscle (*σ_mus_*). These variables were chosen because they are essential for initiation and self-sustaining vocal fold oscillation at a desired fundamental frequency and intensity. Researchers have made attempts to find these relationships using electromyographic recordings in humans [22–26], in-vivo canine larynges experiments [21,27–31], and computational models [32–34]. However, majority of these attempts were made to identify the relationship with fundamental frequency only and to a lesser extent with vocal intensity [35,36]. In this study, we use computational tools to extend to all the four acoustic and two postural features. Additionally, we compare the results from this study with results published in some previous studies.

The earlier works of electromyography recordings in humans was done by Faaborg-Andersen (1957, 1965) [22,23], Hirano et al. (1969) [24], and Gay et al. (1972) [25]. Hirano et al. (1969) recorded the electromyography activity of three intrinsic laryngeal muscles in the regulation of fundamental frequency and vocal intensity in six subjects. The muscles studied were CT, IA, and the vocalis portion of the TA muscles. The results suggested that all three muscles were involved in regulating fundamental frequency and intensity, with *f_o_* and intensity increasing with an increase in CT activation. Gay et al. (1972) also found that CT plays a major role among all the five intrinsic laryngeal muscles in the control of fundamental frequency, and subglottal pressure in the control of intensity. With electromyography conducted on four subjects, Titze et al. (1989) [32] found that *f_o_* can both increase and decrease with increased TA activity, but only increases with CT activity. More recent studies in humans focused on intrinsic laryngeal muscle coordination during normal speech, respiration, and swallowing [37–39]. Hillel, (2001) [38] found that TA and LCA activity decreased after initial burst preceding phonation onset, while the IA sustained glottis position during phonation.

Animal model studies on muscle activations predominantly focused on canines because their vocal fold physiology is close to that of humans. Earlier in vivo canine model studies used electrical stimulation to study the function of IA [27,28] and PCA [29] muscles during phonation. The IA was thought to mainly control the amount of subglottic pressure available during phonation. Recent studies using in vivo canine models also used electrical stimulation but with much finer control to investigate the effect of intrinsic laryngeal muscle activation on the fundamental frequency and glottal posture [30], as well as on fundamental frequency and intensity [31]. The muscles were activated independently from threshold to maximal contraction while airflow was increased to phonation onset and beyond. It was observed that onset frequency was primarily affected by CT activation, and TA increased or decreased *f_o_* and sound pressure level (SPL) under other muscle activation conditions. The LCA/IA activity maintained vocal fold adduction at higher subglottic pressure levels. Sound pressure level was highly correlated with subglottal pressure in all conditions. They also found that the same *f_o_* and SPL could be achieved with a variety of muscle activation combinations, as reported earlier in [32].

Computational models have been used to study the effects of intrinsic laryngeal muscle activations on fundamental frequency and intensity. Farley (1996) [33] used a simplified mathematical model of the larynx to find that *f_o_* is simultaneously controlled by TA, cricothyroid pars oblique (CTO), and cricothyroid pars recta (CTR). Titze and Story (2002) [34] modeled CT, TA, LCA, and PCA muscle activations along with lung pressure to control a three-mass vocal fold model. The results showed that oscillation regions in muscle activation control spaces are similar to those measured in human subjects. Lowell and Story (2006) [40] also used a three-mass vocal fold model for voice simulation in adult males to study the role of individual CT and TA muscle activities on *f_o_*. They found that largest *f_o_* change with CT activation was observed at low TA levels.

Coming to the posturing features, Chhetri et al. (2010) [21] used an in vivo canine model to study the role of intrinsic laryngeal muscle activations on vocal fold length. At lower superior laryngeal nerve (SLN) stimulation levels, they observed linear change in length, and at higher levels, the strain reached a plateau. Chhetri et al. (2012) [30] also included glottal distance at the vocal processes as one of the posturing features. They observed that LCA/IA activation primarily closed the cartilaginous glottis and TA activation closed the mid-membranous glottis. Vahabzadeh-Hagh et al. (2017) [41] used an in vivo canine hemilarynx model to study the role of paired intrinsic laryngeal muscles on three-dimensional vocal fold postural changes. They found that combined TA and CT activation yields a rectangular glottal surface, and further addition of TA yields a divergent glottis.

The current study focuses on the role that TA and CT muscle activations play in controlling the four acoustic variables (*f_o_*, SPL, spectral content, and aperiodicity) and two postural variables (length and tissue fiber stress in the three vocal fold layers). To the best of our knowledge, there is no previous study that focused on the combination of these variables for self-sustained vocal fold oscillation in a finite-element computational model. In this study, we used the fiber-gel finite element model, a muscle activation-based model that produces flow-induced oscillation. The model accepts five intrinsic laryngeal muscle pairs as inputs to set up prephonatory glottal shapes. Lung pressure then drives the vocal folds into self-sustained oscillation. The outputs of the model are many time-varying signals, including oral pressure, oral flow, glottal flow, glottal flow derivative, and transglottal pressure. The findings from the fiber-gel model are compared to the results from human electromyography (EMG) studies (Titze et al. 1989) [32], and in vivo canine model studies (Chhetri et al. (2012) and Chhetri and Park, 2016) [30,31].

## 2. Materials and Methods

### 2.1. Fiber-Gel Finite Element Model

**Figure 1.**
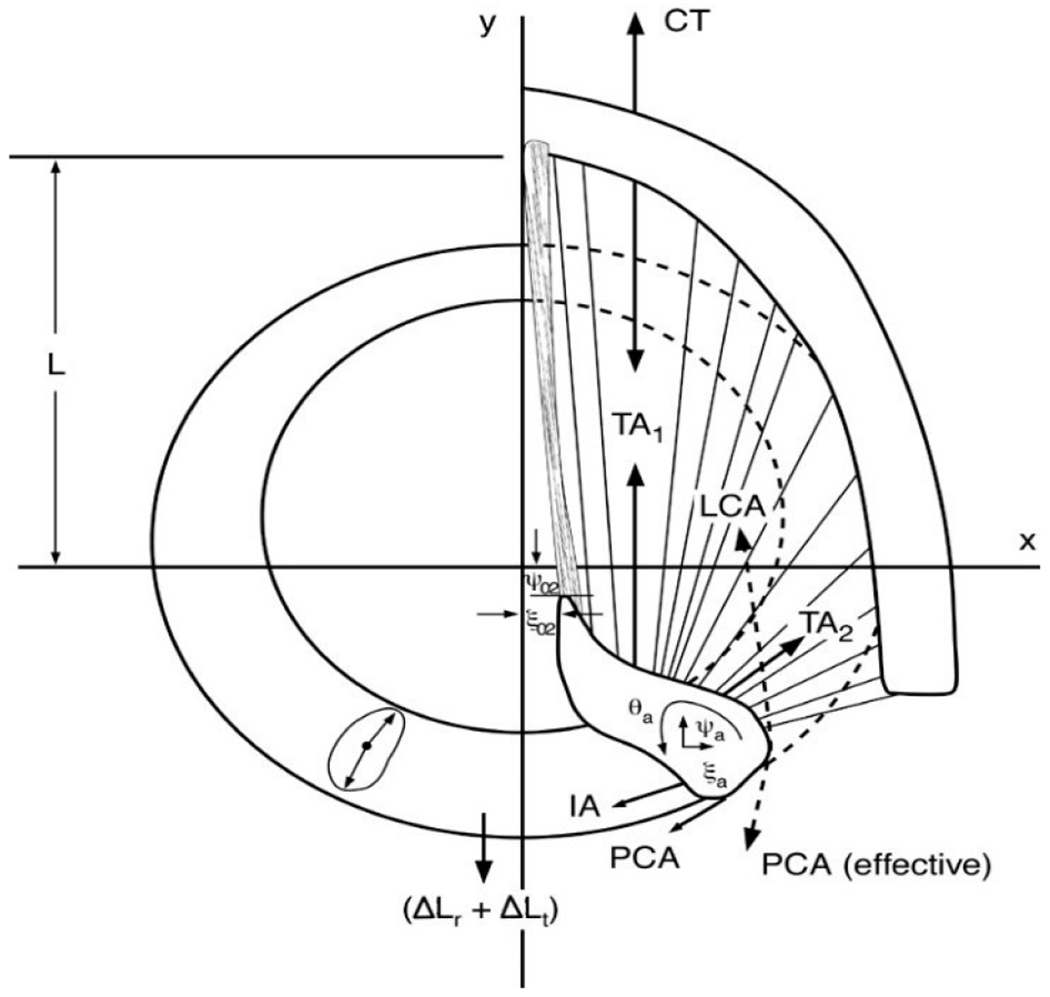
Forces acting on arytenoid cartilage for cricoarytenoid movements (Titze, 2006 [43]).

The fiber-gel model combines a viscoelastic ground substance (a gel) with directional fibers under tension in all layers of the vocal fold (superficial layer of the lamina propria (SLLP), ligament, and muscle) [42]. At the physiologic level of input, the control parameters are lung pressure and activation levels of five intrinsic laryngeal muscle pairs (if left-right symmetry is assumed), CT, TA, LCA, PCA, and IA. With these inputs, and stored anatomical dimensions of the larynx ([43], Chapter. 1) and the airways [44], pre-phonatory vocal fold and vocal tract shapes are produced. Figure 1 shows how all five intrinsic muscles are contributing to the vocal fold length, *L* and the position (ξ_02_, ψ_02_) of the vocal process of the arytenoid cartilage in the horizontal plane. The plane is at the level of the vocal ligament. All muscles except the CT apply forces directly to the arytenoid cartilage. The CT has a forward and downward pull on the thyroid cartilage relative to the cricoid cartilage, therewith elongating the vocal folds. The dotted-line vector for LCA indicates that the fibers run below those of the TA, while the dotted line for the PCA vector indicate an effective force direction due to curvilinear fiber attachment. The combined mechanics of the cartilages is solved with the second-order Runge-Kutta differential method [43]. Only right-side cartilages and forces were shown in Figure 1 for clarity. The mechanics of the cartilages on the left side are solved similarly.

In the rostral-cranial (vertical) direction, the medial surface of the vocal folds is defined with three parameters, the vocal fold thickness *T*, the lower adduction of the arytenoid cartilage *ξ*_01_, and a medial surface bulging factor *ξ_b_*. With these parameters, the glottal half-width at any point (*y, z*) along the surface of each vocal fold is defined as

(1) $$\xi_{0}(y,z)=(1-y/L)\left[\xi_{02}+\left(\xi_{01}-\xi_{02}-4\xi_{b}z/T\right)(1-z/T)\right]$$

**Figure 2.**
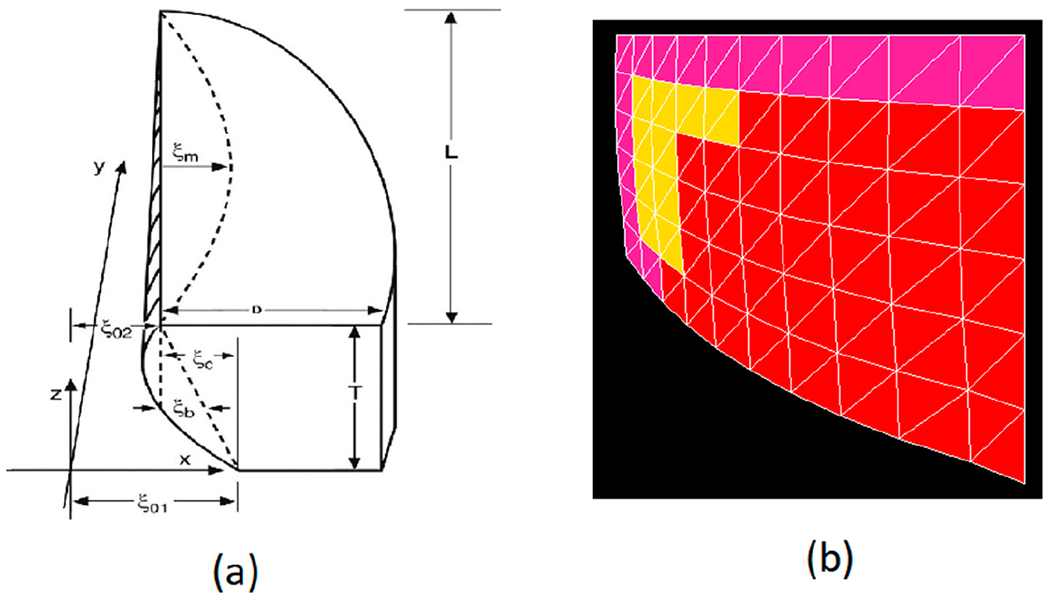
(**a**) Right vocal fold geometry in the Fiber-Gel Model, and (**b**) coronal section of the right vocal fold in finite element implementation. Purple is the superficial layer of the lamina propria (SLLP), yellow is the ligament, and red is the thyroarytenoid (TA) muscle.

The parameters in these equations are determined by muscle activations with empirical rules

(2) $$L=L_{0}(1+\varepsilon )$$

(3) $$T=T_{0}/(1+\varepsilon )$$

(4) $$\xi_{01}=\xi_{02}+\max\left(0,0.1T\left(1-1.5a_{TA}\right)\right)$$

(5) $$\xi_{b}=0.05T\left(1-a_{TA}\right)$$

where *ε* is the vocal fold strain (determined by all muscles) and *a_TA_* is the activation of the TA muscle. Here, *L*_0_ was set to be 1.6 cm, and *T*_0_ was set to be 0.7 cm for an average male vocal fold. Figure 2a shows the right vocal fold geometry as parameterized by the empirical rules given above. Also shown is the variable *ξ_m_*, the amplitude of vibration when self-sustained oscillation takes place. The left vocal fold is also governed by the same parameters. The geometric properties of the layers are modeled according to the histological data available from the literature [45–47]. The full details of vocal fold posturing with intrinsic laryngeal muscle activations is provided in ([43], Chapter 3).

As tissue is deformed with muscle contraction (e.g., length, thickness, and medial surface of the vocal folds), viscoelastic parameters are modified with basic constitutive equations. The passive fibrous tissue properties in the y-direction in the three vocal fold layers and the other intrinsic laryngeal muscles are characterized with a combined linear and exponential stress function ([43], p. 77):

(6) $$\sigma_{y}=-\sigma_{0}\left(\varepsilon -\varepsilon_{1}\right)+\sigma_{2}\left[e^{B\left(\varepsilon -\varepsilon_{2}\right)}-1-B\left(\varepsilon -\varepsilon_{2}\right)\right]$$

In the above equation, *σ*_0_ and *σ*_2_ are scale factors, *ε*_1_ is the strain where the linear function begins and *ε*_2_ is the strain where the exponential term begins. The stress *σ_y_* is converted into an equivalent shear modulus μ′, to which a gel shear modulus μ is added for transversally isotropic gel properties. In combination, the viscoelastic model becomes a fiber-gel model. The active properties of all intrinsic muscles are determined by a modified Kelvin model ([43], p. 76). The modified Kelvin model is governed by maximum isometric active stress, *σ_m_*. Table 1 shows the parameters of Equation (6) and *σ_m_* used in this study. The full details of the viscoelastic properties obtained from measurements on multiple species’ biological tissues are given in ([43], Chapter 2).

Each vocal fold consisted of three layers: SLLP, vocal ligament, and TA muscle. A two-dimensional finite-element mesh as seen in Figure 2b was constructed for each of *M* = 15 coronal slices in the posterior-anterior direction of each vocal fold, following [48]. The elements were triangular, allowing the shape functions (interpolations within the elements) to be computed analytically from the nodal points. This choice was based on speed of computation. There were two triangular elements per cell, with *L* = 10 columns of cells and *N* = 6 rows of cells in the coronal plane ([43], pp. 214–231). In solving the matrix equations for (L+1)*(N+1) nodal displacements, aerodynamic forces were applied at the open boundaries and string (fiber) forces were applied between the slices. The material in each slice was considered incompressible and transversally isotropic [48]. Thus, one elastic constant (the transverse shear modulus *μ* = 0.5 kPa for all the layers) and a viscosity *η* (= 0.1 Pa-s for all the layers) defined the gel property. A second shear modulus *μ*′ defined the fiber property (Equation (6) above). Boundaries were fixed at anterior, lateral, and posterior surfaces. Boundaries were free to move with pressure distributions on inferior, medial, and superior surfaces. The lateral boundary was curved as shown in Figure 2a.

The vibrational displacement was superimposed on the postural displacement. A Bernoulli approach was used to calculate glottal pressures up to the minimum glottal diameter, from which point jet flow was assumed in the remainder of the glottis ([43], Chapter 7). When lung pressure is turned on, airflow and surface pressures are computed in the glottis (air space between the vocal folds) and on all surfaces along the vocal tract using wave-reflection algorithm [43,49,50]. The vocal tract airways were spatially sampled with sections of 0.3968 cm length, half the distance sound travels in 1/44,100 s. There were 36 subglottal sections and 44 supraglottal sections. The choice of section length allowed forward and backward travelling waves to be computed at section boundaries at a sampling rate of 44.1 kHz, in synchrony with wave propagation to guarantee computational stability. Energy dissipation was included by incorporating viscous losses and yielding wall losses within each section, as well as kinetic losses at section boundaries [51]. Radiation from the mouth was computed with a low-frequency parallel inertance/resistance equivalent of the radiation impedance ([43], Chapter 6, [52]).

The vocal folds will self-sustain oscillation if vocal fold adduction, glottal geometry, and elastic and viscous properties are such that phonation threshold pressure (PTP) is exceeded with the applied lung pressure. The oscillation modulates the airflow, producing oscillatory flow and acoustic pressures throughout the airways. The basic vibrational and acoustic underpinnings for the fiber-gel finite element model are detailed in Titze (2006 [43], Chapters 6 and 7)). More recent applications and validations have been published in [51,53,54].

### 2.2. Data Collection

A brute force approach was used to vary TA and CT muscle activations. TA and CT muscle activations were varied between 0% and 100% of their maximum muscle activation levels in steps of 5%. Lung pressure, LCA, PCA, and IA activations were initially also varied randomly for the given CT and TA activations, but ultimately needed to be constrained to focus only on cases of self-sustained oscillation (a small percentage of the initial exploratory set). The constraint equations are given in the Results section. A total of 441 signals were generated using this approach. Each signal was 0.4 s in duration. The waveforms, oral pressure (*p_o_*), vocal fold length (*L*), and fiber stress (*σ_y_*, see Equation (6)) in the three vocal fold layers (SLLP, vocal ligament, and TA muscle) were recorded for every signal. Using these waveforms, acoustic and postural features were computed. Fundamental frequency was computed using the SWIPE’ algorithm [6]. Sound pressure level radiated at 30 cm from mouth was computed using the following equation [7]:

(7) $$SPL=10\log_{10}\frac{I}{I_{0}}=10\log_{10}\frac{{\left({\bar{p}}_{o}\right)}^{2}}{4\pi R^{2}R_{m}I_{0}}$$

where the bar denotes a time-average of the oral pressure, *p_o_, R* is the radius from mouth, *R_m_* is radiation resistance [52,55], and *I*_0_ is the standard reference intensity (10^−12^ watt/m^2^).

Normalized spectral centroid was computed to quantify brightness [9]. Spectral centroid (SC) measures the center of mass of the spectrum in Hz. It is computed using the following equation:

(8) $$SC=\frac{\sum_{k=0}^{N}f_{k}x_{k}}{\sum_{k=0}^{N}x_{k}}$$

Here, *N* is total number of frequency bins, *f_k_* is the center frequency of the bin in Hz, and *x_k_* is the spectral magnitude in bin *k*. The normalized spectral centroid was then computed by dividing the spectral centroid by the fundamental frequency of the signal.

Approximate entropy (ApEn) quantifies the amount of irregularity and unpredictability present in a signal [56]. For a given time series signal *x*(*t*), an m-dimensional delay-coordinate phase space *X* = {*x*(*t*), *x*(*t* − *τ*),… , *x*(*t* − (*m* − 1)*τ*)} was first constructed [18]. Here, *m* is the embedding dimension and *τ* is the time delay. Then, ApEn can be computed using the following equation:

(9) $$ApEn(m,r)=\underset{N\to \infty }{\lim}\left[{\text{Φ}}^{m}(r)-{\text{Φ}}^{m+1}(r)\right]$$

where

(10) $${\text{Φ}}^{m}(r)={\left(N-m+1\right)}^{-1}\overset{N-m+1}{\underset{i=1}{\sum^{\text{​}}}}\log C_{i}^{m}(r)$$

Here, $C_{i}^{m}\left(r\right)$ is the correlation integral computed as suggested in [56], *r* is the radius of similarity (chosen as 0.2 *variance(*x*)), and *N* is the number of samples of the signal *x*(*t*). In the current study, *m* was set to 2, and *τ* was set to 1 for all the signals.

## 3. Results

### 3.1. Lung Pressure

Only the cases for which phonation threshold pressure was exceeded were generated. To guarantee these conditions, lung pressure was increased with both CT activation and TA activation according to the following relation:

(11) $$P_{L}=0.8+0.025a_{CT}+0.01a_{TA}\text{kPa}$$

where *a_CT_* and *a_TA_* are normalized activation levels ranging from 0% to 100%.

**Figure 3.**
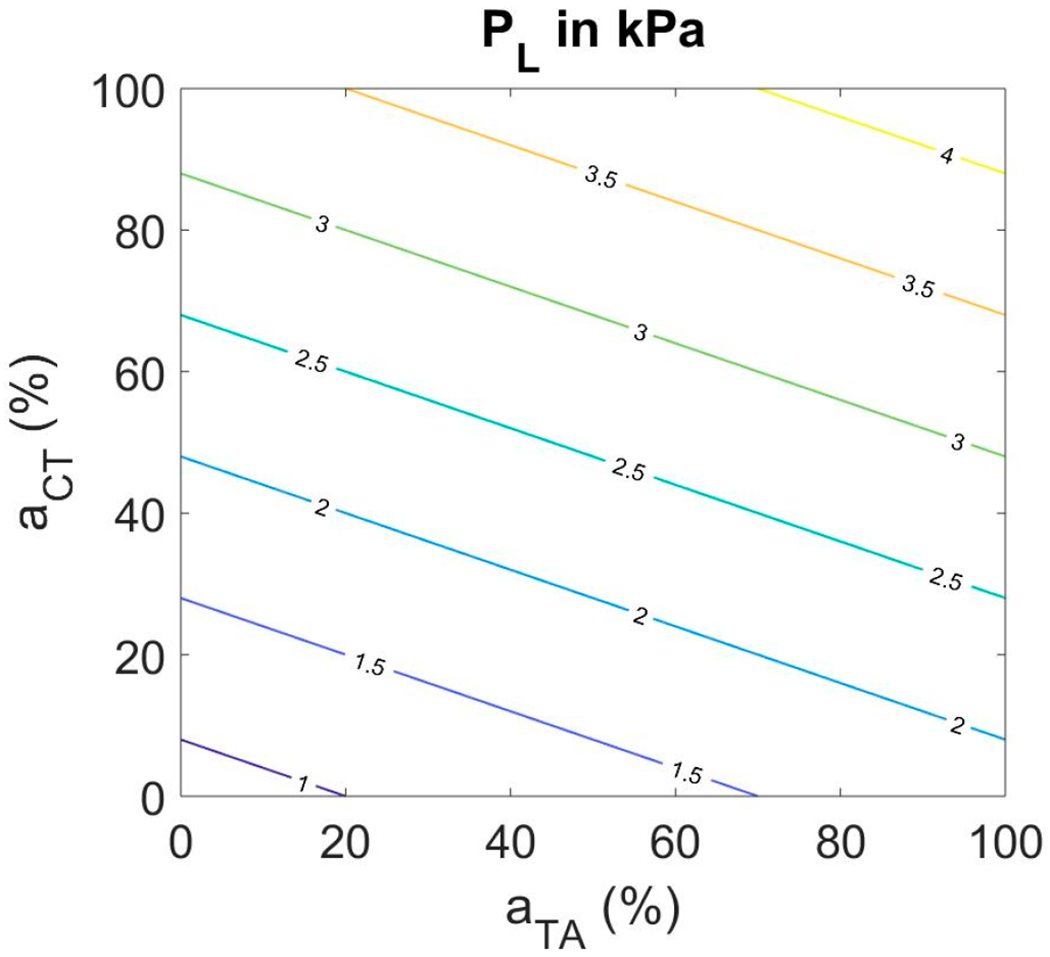
Muscle activation plot with lung pressure as the contour parameter.

The increase with CT activation needed to be greater than the increase with TA activation to remain above threshold for self-sustained oscillation. The equation results in a range of 0.8 kPa and 4.3 kPa for the lung pressure. Figure 3 shows a muscle activation plot with lung pressure as the contour parameter.

Note that lung pressures up to 4.3 kPa are needed in this model to obtain self-sustained oscillation, but we do not claim that the simple linear relation in Equation (9) represents threshold pressure or an equal fraction above threshold.

### 3.2. Muscle Activation for Adduction

**Figure 4.**
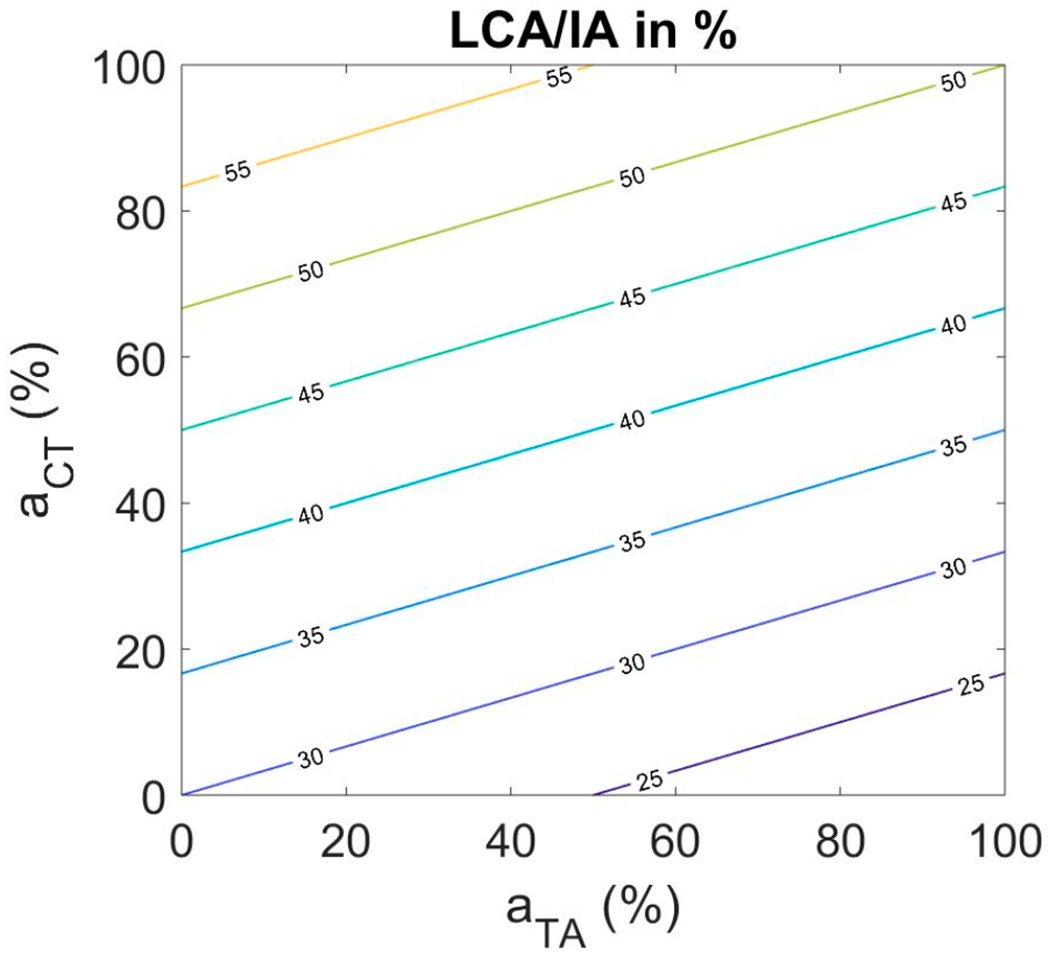
Muscle activation plot with LCA/IA activation as contour parameter.

To guarantee self-sustained oscillation, LCA and IA activation needed to be kept in a relatively smaller range. Phonation threshold pressure is highly sensitive to vocal fold adduction [57]. The following relation was used:

(12) $$a_{LC}=a_{IA}=30+0.3a_{CT}-0.1a_{TA}$$

where *a_LC_* is the activation level of the LCA muscle normalized to its maximum value for forceful adduction and *a_IA_* is the activation level of the IA muscle normalized to its maximum value. Figure 4 shows the muscle activation plot with the balanced LCA/IA combination as the contour parameter. A larger increase in LCA/IA activation with CT activation was needed than with TA activation because tissue incompressibility demands that the vocal folds abduct slightly when they are elongated. By contrast, TA activation shortens the vocal folds and adducts them slightly. Note that, overall, the LCA/IA activation range for self-sustained oscillation is between 30% and about 60% over the entire range of TA and CT activations.

Variations with posterior cricoarytenoid (PCA) activation were not included in this first attempt to address the mapping from physiologic input to acoustic output. PCA activation was kept constant at 0%. It is understood that the PCA muscle can be active in phonation. It is used for high-pitched phonation to resist the anterior pull of the arytenoid cartilage by the CT muscle. At this stage in the modeling of the laryngeal framework mechanics, we have included this resistance to anterior movement by increasing the stiffness of the cricoarytenoid joint in a nonlinear fashion, thereby limiting anterior movement of the cartilage.

### 3.3. Acoustic Features

Physiologic inputs and control parameters were mapped to the four acoustic features fundamental frequency, sound pressure level, normalized spectral centroid, and approximate entropy. Muscle activation plots were generated for each of the four acoustic features with respect to CT and TA muscle activation levels.

#### 3.3.1. Fundamental Frequency

**Figure 5.**
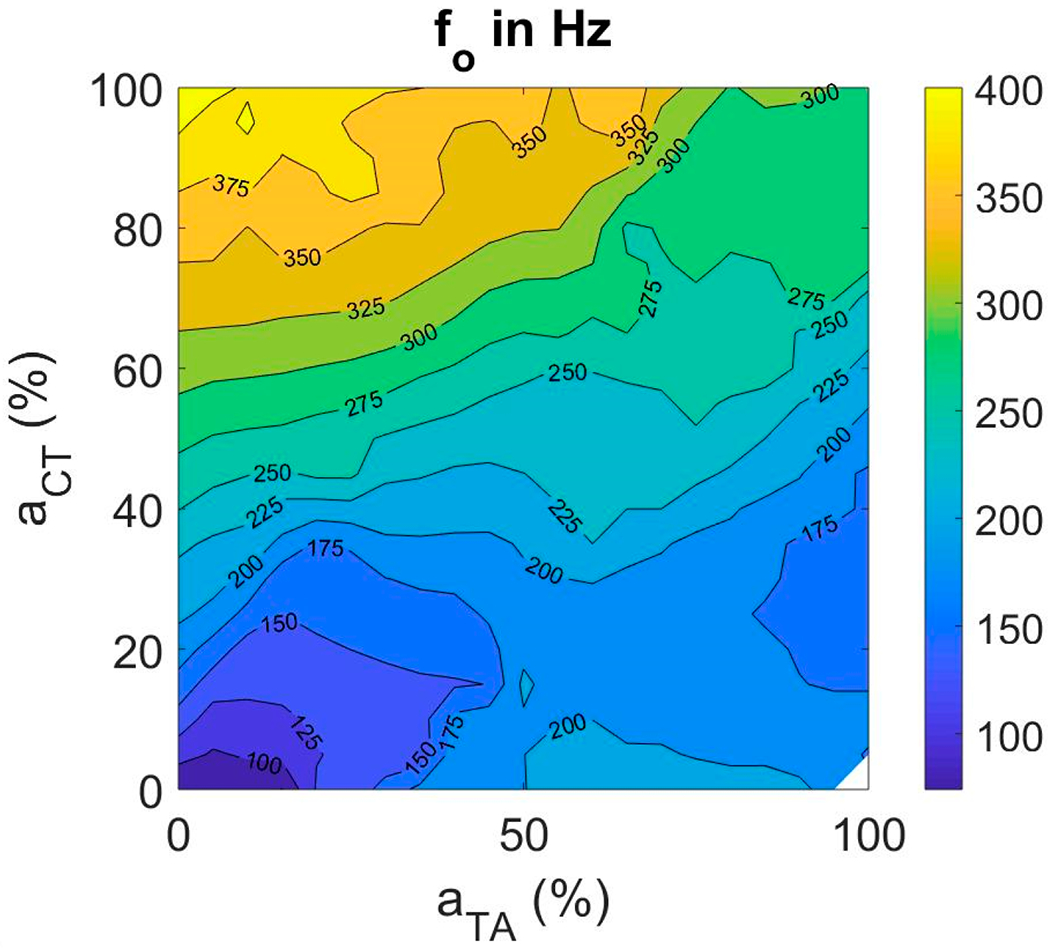
Muscle activation plot with fundamental frequency (*f_o_*) as contour feature.

Fundamental frequency (*f_o_*) increased with increase in CT activity for all TA activation levels (as seen in Figure 5). To the contrary, *f_o_* increased with increase in TA activity at low CT activation levels and decreased with increase in TA activity at high CT activation levels. The range of *f_o_* achieved with the fiber-gel model is between 70 and 400 Hz with anatomical dimensions adjusted for males.

#### 3.3.2. Sound Pressure Level (SPL)

**Figure 6.**
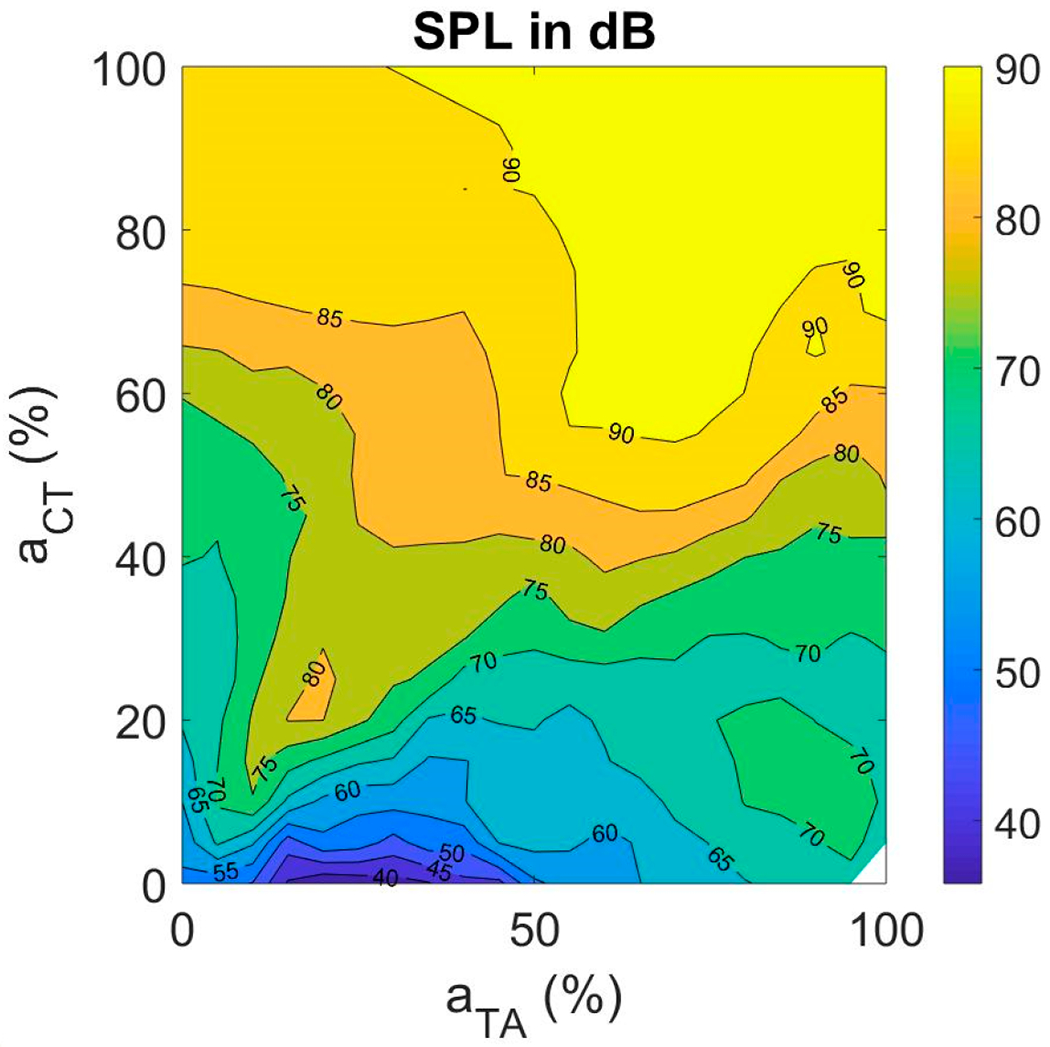
Muscle activation plots with sound pressure level (SPL) as the contour feature.

SPL was found to increase steadily with CT activation (Figure 6). The increase in fundamental frequency with CT is the primary explanation. SPL increases on the order of 6–9 dB with every doubling of *f_o_* [58]. With TA activation, SPL increased uniformly if there is a co-variation with CT activation. In the fiber-gel model described here, SPL varied between 40 and 90 dB across the CT and TA activation levels. It is interesting to note that the steepest assent in SPL is along the diagonal, increasing TA activation in proportion to CT activation.

**Figure 7.**
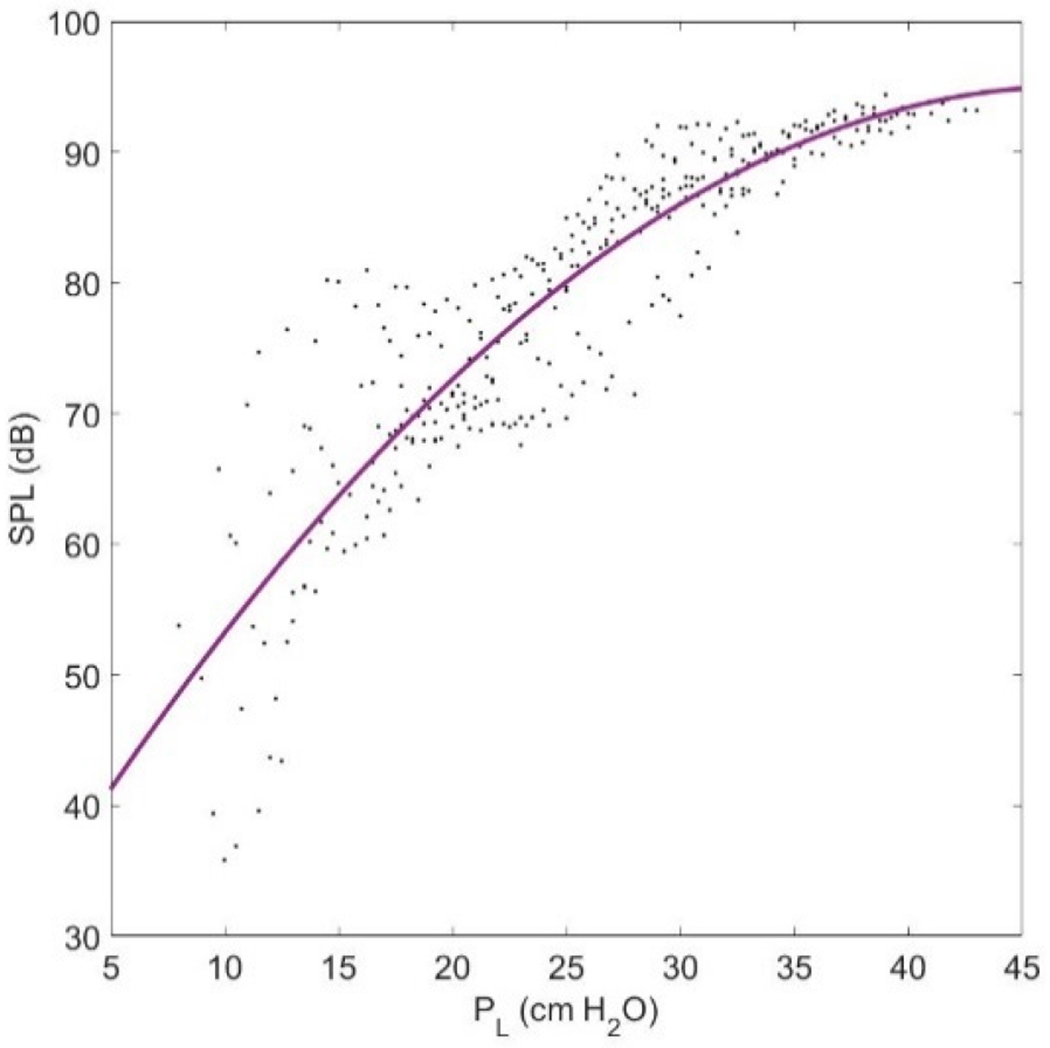
Scatter plot of SPL vs. P_L_ with a quadratic polynomial fit.

As known from previous studies, SPL strongly depends on P_L_ [59]. Figure 7 shows a plot of SPL as a function of P_L_ as computed with the fiber-gel model. The gradual saturation in SPL with lung pressure increase results from a limitation in amplitude of vibration due to vocal fold collision. A quadratic relation between SPL and P_L_ is shown to be a reasonable approximation.

#### 3.3.3. Brightness of a Sound

**Figure 8.**
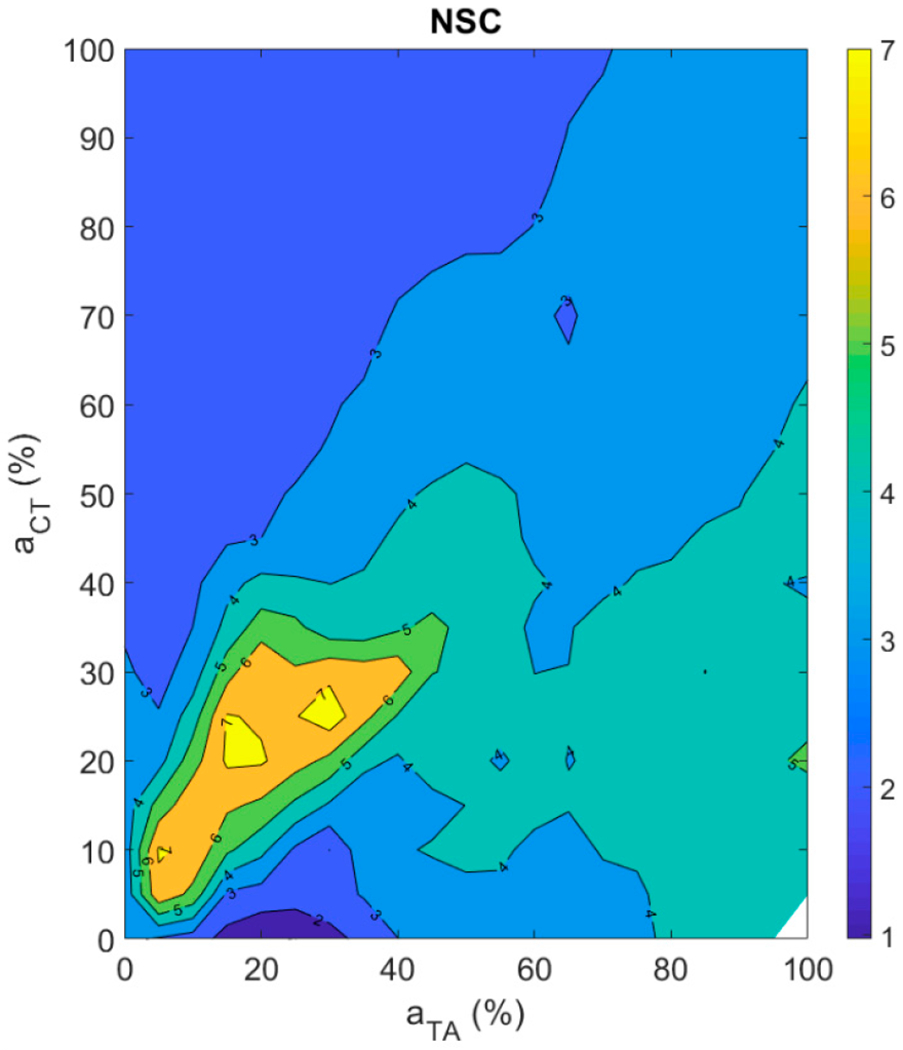
Muscle activation plot with normalized spectral centroid (NSC) as the contour feature.

As suggested in the methods section, brightness of a sound can be represented with the normalized spectral centroid (NSC). Figure 8 shows the muscle activation plot with NSC as the contour feature. The NSC is increasing along the diagonal up to about 20% CT and TA activation levels and is decreasing thereafter. This suggests that about 20% CT and TA activation levels are optimal to obtain high NSC in the fiber-gel model. The NSC is steadily decreasing away from the diagonal with increase in CT activation level. The trend away from diagonal is similar for TA activation but not as strong as is observed with CT activation.

#### 3.3.4. Aperiodicity Roughness in the Vocal Sound

**Figure 9.**
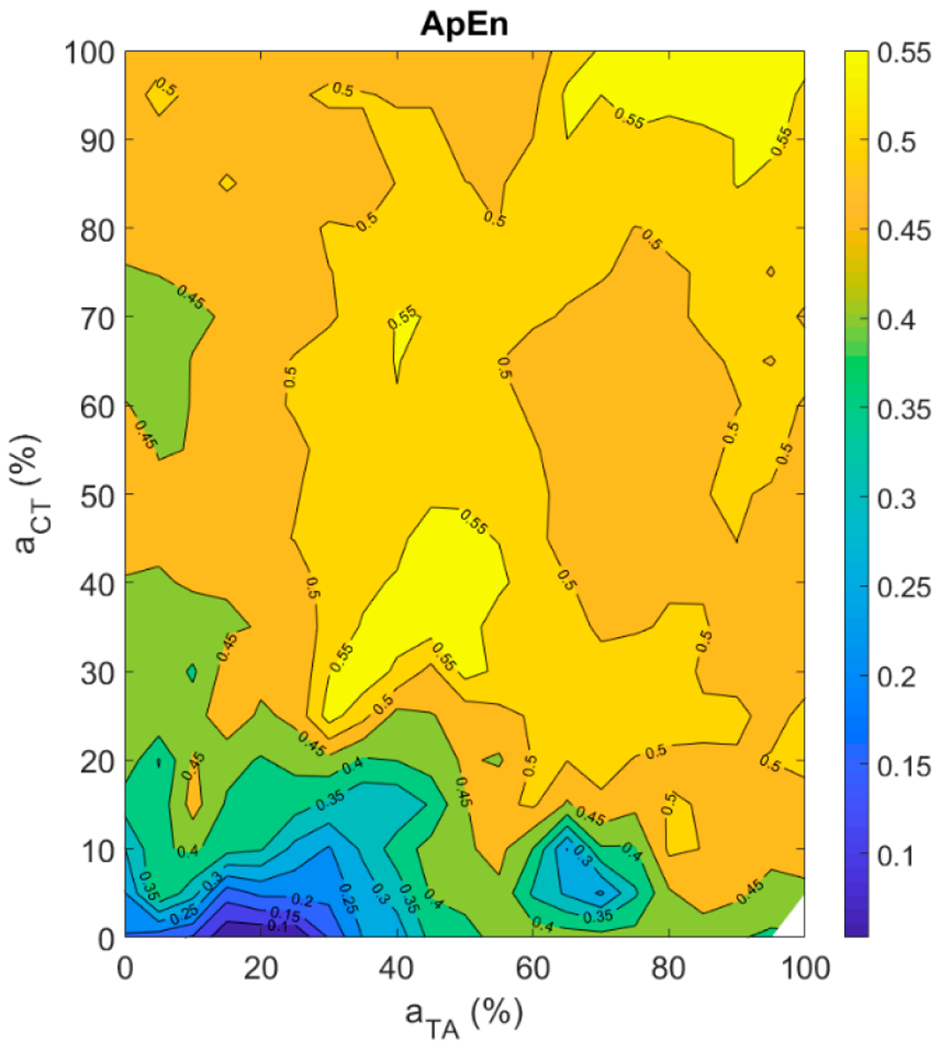
Muscle activation plot with approximate entropy as the contour feature.

Approximate entropy (ApEn) was used to quantify aperiodicity roughness in the vocal sound. The higher the ApEn value, the greater the aperiodicity roughness in a signal [56]. It can be observed from Figure 9 that the smallest ApEn value was obtained at about 20% TA and close to 0% CT activation levels. The maximum ApEn values were obtained on the diagonal at about 40% as well as at 90% CT and TA activation. Overall, periodic signals were obtained at low CT and TA activation levels and aperiodicity in the signals increased as CT or TA activation level was increased. However, the muscle activation plot is still very complex to read due to the presence of several islands of high or low aperiodicity.

### 3.4. Posturing Features

For the postural features, an average over the last 100 ms was computed to obtain the steady state values.

#### 3.4.1. Vocal Fold Length

**Figure 10.**
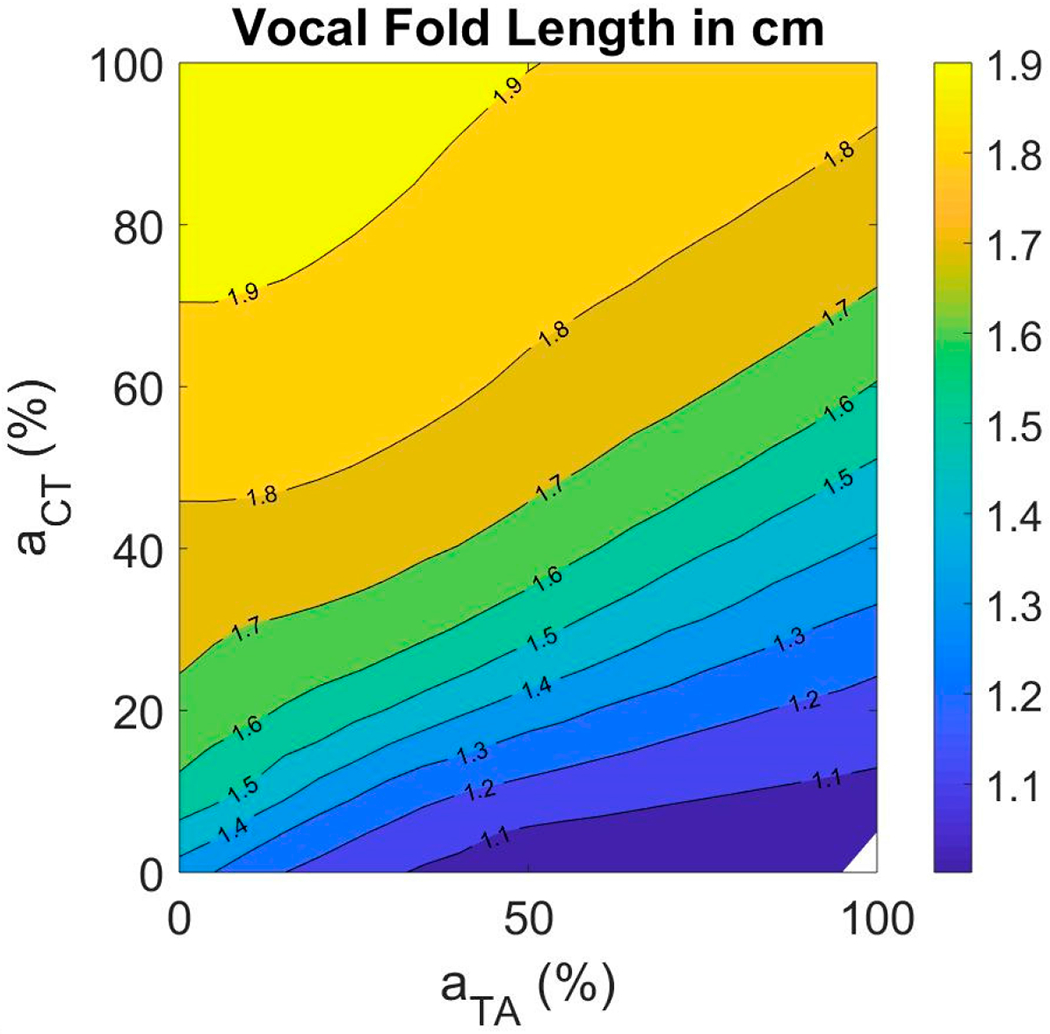
Muscle activation plot with vocal fold length as the contour feature.

Range of vocal fold length is an important indicator of range of motion between the thyroid and cricoid cartilages. It is also predictive of the range of fundamental frequency [60]. The resting length of the vocal folds in the fiber-gel model was 1.6 cm. By varying TA and CT muscle activations between their limits, the vocal fold length varied between 1.0 cm and 1.9 cm (Figure 10). The length increased with increase in CT activation and decreased with increase in TA activation. It can be observed that the length did not increase uniformly with increase in CT activation. This is attributed to a non-linear increase in vocal fold fiber stress in all tissue layers as a function of CT activation.

#### 3.4.2. Fiber Stress in Vocal Fold Layers

**Figure 11.**
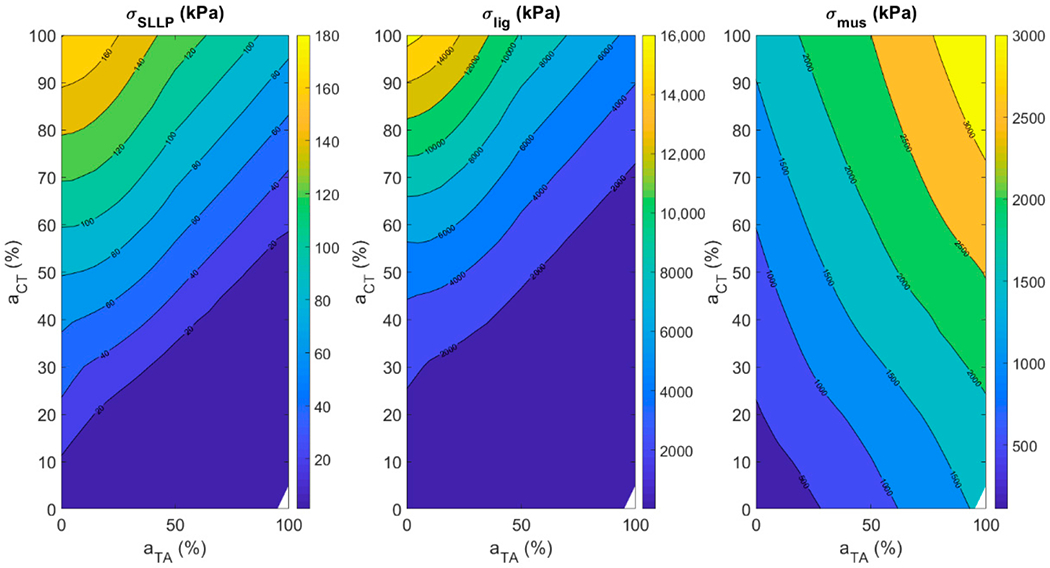
Muscle activation plots for fiber stress in (left) SLLP layer, (center) ligament layer, and (right) TA muscle with respect to TA–CT muscle activations.

Figure 11 shows the muscle activation plots for vocal fold fiber stress with respect to CT–TA muscle activations in all the three vocal fold layers (left for SLLP, center for ligament and right for TA muscle). The fiber stress in the SLLP layer is decreasing with increase in TA activity and increasing with increase in CT activity. The pattern is similar in the case of fiber stress in the ligament layer, but at higher CT activity. At low CT activity, the fiber stress in the ligament was not dependent on TA activity. The fiber stress in the TA muscle layer increases with increase in both CT and TA activity. The vocal fold ligament has the highest fiber stress, followed by TA muscle, followed by SLLP layer. The maximum stress achievable for ligament, TA muscle, and SLLP are in the ratio 88.8:16.6:1.

## 4. Discussion and Conclusions

The current study focused on how TA and CT muscle activation levels control various acoustic and posturing features of voice production. Lung pressure, LCA, and IA activation levels were varied as a function of TA and CT activation levels such that the phonation threshold pressure was always exceeded. PCA muscle activation was set to 0%. The results suggested that *f_o_* and SPL increase with CT activation, while NSC decreases when CT activation is raised above 20%. With TA activation, acoustic features have no uniform trends, except that SPL increases uniformly with TA if there is a co-variation with CT activation. The postural features L, *σ_SLLP_, σ_lig_*, and *σ_mus_* increase with an increase in CT activation. Also, *σ_mus_* increase whereas L, *σ_SLLP_*, and *σ_lig_* decrease with an increase in TA activation.

Aperiodicity roughness was found to be the most complex feature to quantify among all the acoustic and postural features that were studied in the current study. In the current study, signal-to-noise ratio, harmonic-to-noise ratio, smoothed cepstral peak prominence [15,16], correlation dimension [18], sample entropy [19], and approximate entropy were tried to appropriately quantify aperiodicity roughness in a signal. None of these acoustic features was completely accurate in measuring roughness in the signals generated by varying TA and CT muscle activations. Approximate entropy was found to be the best among them, as judged by visual inspection of signal aperiodicity and tests on sinusoidal signals with variable amounts of roughness. However, even ApEn does not give smooth contours in the muscle activation plots, suggesting that further research needs to be conducted to obtain an acoustic feature that can accurately quantify aperiodicity in a voice signal.

Comparing our results with findings from previous studies sheds more light on the relationship between input and output variables. Titze et al., (1989) [32] used electromyography on four subjects to study the role of CT and TA muscle activations in the regulation of fundamental frequency of phonation. Similar to the current findings, *f_o_* increased with increase in CT activation at low TA values. There was also a decrease in *f_o_* with increase in TA activation at high CT levels. No phonation was produced at extremely high TA and low CT by these subjects. They were not asked to produce anything higher than 440 Hz. We observed phonation above 440 Hz in our model, but the data were sparse with relatively large lung pressures and more irregularity.

It is well known that subglottal pressure plays a major role in the control of intensity. Our study confirmed this. Aside from P_L_, CT activation had a major impact on SPL. This is probably due to the increased radiation efficiency with higher *f_o_* [52]. Every doubling of *f_o_* adds at least 6 dB to the radiated sound pressure level.

No prior formal study has related a brightness feature like the normalized spectral centroid (NSC), or a roughness feature like approximate entropy (ApEn), to intrinsic laryngeal muscle activations. Hence, we could not compare our results against findings from other studies. Relations obtained for NSC and ApEn were much more complex than those obtained for *f_o_* and SPL. Qualitatively, periodicity is related to the dominance of a single mode of vibration and entrainment of other modes to this dominant mode. When the ligament and the TA muscle fibers do not have similar natural mode frequencies, there is often not the required entrainment. This leads to aperiodic vibration. Such rough phonation was observed in many regions of the muscle activation plot.

With regard to the posturing features, vocal fold length change (strain) was found by Chhetri et al., (2012) [30] to change linearly with superior laryngeal nerve stimulation at low levels and reached a plateau at high stimulation levels. Vocal fold strain also decreased linearly with recurrent laryngeal nerve stimulation amplitude [21]. This result was also observed in our study. The vocal fold length increased linearly at low CT muscle activation levels and reached a plateau at high activation levels. The length decreased with increase in TA muscle activation at all CT activation levels. Vocal fold fiber stress was quantified in terms of muscle activations for the three layers of the vocal folds. All stresses increase exponentially with CT activation. For TA activation, fiber stresses decrease for SLLP and ligament, but increase for TA muscle fibers. This result correlates with vocal fold length change.

It should be noted that the conclusions from the current study are dependent on the accuracy of fiber-gel posturing model, which is not yet fully developed for three-dimensional vocal fold deformation with muscle activation. In particular, the medial surface bulging with TA contraction is currently being investigated in detail. Future investigations should be validated with further human and animal electromyography experiments, especially for the brightness and roughness features.

## Figures and Tables

**Table 1.** Parameter values used in fiber-gel finite element model for modeling the five intrinsic laryngeal muscles (CT, LCA, TA, IA, and PCA) along with vocal ligament (LIG) and SLLP layers.

	CT	LCA	TA	IA	PCA	LIG	SLLP
*σ*_0_ (dyn/cm^2^)	2.2 × 10^4^	3 × 10^4^	2 × 10^4^	2 × 10^4^	5 × 10^4^	2 × 10^4^	.2 × 10^4^
*σ*_2_ (dyn/cm^2^)	5× 10^4^	59× 10^4^	1.5× 10^4^	30× 10^4^	55× 10^4^	.15 × 10^4^	1.5 × 10^4^
B	7	4	6.5	3.5	5.3	12.8	6.5
*ε*_1_	−0.9	−0.9	−0.9	−0.9	−0.9	−0.9	−0.9
*ε*_2_	−0.06	0.05	−0.5	0	0.1	−0.5	−0.2
*σ_m_* (dyn/cm^2^)	400× 10^4^	140× 10^4^	180× 10^4^	140× 10^4^	96× 10^4^		

CT-cricothyroid; LCA-lateral cricothyroid; TA-thyroarytenoid; IA-interarytenoid; PCA-posterior cricoarytenoid; SLLP-superficial layer of lamina propria.

## Featured Application:

The goal of this research publication is to advance the current state of computer simulation of voice production. Many future questions about the physiology, biomechanics, and acoustics of vocalization are expected to be answered by simulation to avoid excessive use of animals and human subjects in experimentation. Here we feature the control of vocal output with muscle activation as inputs, a particularly difficult experimental procedure to conduct on animals and human volunteers.
